# 

**Published:** 1894-02

**Authors:** 


### TABLE OF CONTENTS.

ORIGINAL COMMUNICATIONS. PAGE

Injurious Effects of Amalgam Fillings. By CHARLES H. TAFT, A.B., D.M.D., Chicago . . . . . . . . . . . . . . . . . . . . . . . . . . 81

The Psychological Aspect of Hypnosis. By WM. ROMAINE NEWBOLD, PH.D. . . . . . . . . . . . . . . . . . . . . . . . . . . . . . . . 92

REPORTS OF SOCIETY MEETINGS.

New York Odontological Society . . . . . . . . . . . . . . . . . . . . 105

Harvard Odontological Society . . . . . . . . . . . . . . . . . . . . 118

EDITORIAL.

Are our Dental Organizations Satisfactory? . . . . . . . . . . . . 129

Dr. Harlan retires . . . . . . . . . . . . . . . . . . . . . . . . . . 133

BIBLIOGRAPHY . . . . . . . . . . . . . . . . . . . . . . . . . . . . . 133

OBITUARY.

Dr. Daniel Neall . . . . . . . . . . . . . . . . . . . . . . . . . . 135

NOTES AND COMMENTS . . . . . . . . . . . . . . . . . . . . . . . . 137

CURRENT NEWS.

Recent Patents . . . . . . . . . . . . . . . . . . . . . . . . . . . 140

Woman's Dental Association of the United States . . . . . . . . . . 140

Dental Commissioners of Connecticut . . . . . . . . . . . . . . . . 141

St. Louis Dental Society . . . . . . . . . . . . . . . . . . . . . . 141

SELECTIONS.

Formaline: An Ideal Harmless Germicide . . . . . . . . . . . . . . 142

Resection of the Inferior Maxilla with Immediate Prosthesis of an Artificial Jaw . . . . . . . . . . . . . . . . . . . . . . . . . . . . 143

